# Self-nanoemulsifying Delivery of Andrographolide: Ameliorating Islet Beta Cells and Inhibiting Adipocyte Differentiation

**DOI:** 10.34172/apb.2021.018

**Published:** 2020-11-07

**Authors:** Yandi Syukri, Muhammad Taher, Ronny Martien, Endang Lukitaningsih, Agung Endro Nugroho, Zainul Amiruddin Zakaria

**Affiliations:** ^1^Department of Pharmacy, Islamic University of Indonesia, Yogyakarta, 55584, Indonesia.; ^2^Department of Pharmaceutical Technology, Faculty of Pharmacy, International Islamic University Malaysia, Bandar Indera Mahkota, 25200, Kuantan, Pahang, Malaysia.; ^3^Faculty of Pharmacy, Gadjah Mada University, Yogyakarta, 55281 Indonesia.; ^4^Department of Biomedical Science, Faculty of Medicine and Health Sciences, Universiti Putra Malaysia, 43400 UPM Serdang, Selangor, Malaysia.

**Keywords:** Adipocyte differentiation, Andrographolide, Self-nanoemulsifying, Pancreatic beta cells

## Abstract

***Purpose:*** Insulin resistance is a characteristic of non-insulin-dependent diabetes mellitus associated with obesity and caused by the failure of pancreatic beta cells to secrete sufficient amount of insulin. Andrographolide (AND) improves beta-cell reconstruction and inhibits fat-cell formation. This research aimed to improve the delivery of water-insoluble AND in self-nanoemulsifying (ASNE) formulation, tested in streptozotocin (STZ)-induced diabetic rats and 3T3-L1 preadipocyte cells.

***Methods:*** A conventional formulation of AND in suspension was used as a control. The experimental rats were orally administered with self-nanoemulsifying (SNE) and suspension of AND for 8 days. Measurements were performed to evaluate blood glucose levels in preprandial and postprandial conditions. Immunohistochemistry was used to assess the process of islet beta cell reconstruction. In vitro study was performed using cell viability and adipocyte differentiation assay to determine the delivery of AND in the formulation.

***Results:*** ASNE lowered blood glucose levels (day 4) faster than AND suspension (day 6). The histological testing showed that ASNE could regenerate pancreatic beta cells. Therefore, ASNE ameliorated pancreatic beta cells. The in vitro evaluation indicated the inhibition of adipocyte differentiation by both AND and ASNE, which occurred in a time-dependent manner. ASNE formulation had better delivery than AND.

***Conclusion:*** ASNE could improve the antidiabetic activity by lowering blood glucose levels, enhancing pancreatic beta cells, and inhibiting lipid formation in adipocyte cells.

## Introduction

This study is a continuation of our previous research on increasing the dissolution and bioavailability of andrographolide self-nanoemulsifying (ASNE). The previous study showed that ASNE (15 mg/mL) demonstrated 12.0 ± 0.2 nm, -46.3 ± 4.2 mV, and 99.4% of particle size, zeta potential, and transmittance, respectively. This formulation is thermodynamically stable and significantly improved dissolution as opposed to andrographolide (AND) suspension. The ASNE also improves the AND bioavailability in rabbit plasma with AUC value enhanced by 1.2-fold, C_max_ increased by 1.26-fold, and Tmax decreased by 1.72-fold when compared to AND suspension.^1^

We expect to examine the correlation between an increase in pharmacokinetic profiles previously studied and the antidiabetic activity of ASNE. Diabetes mellitus (DM) is a common metabolic disorder characterized by chronic hyperglycemia due to the effects of insulin secretion or insulin action. It currently affects 463 million people worldwide but this figure is estimated to reach up to 578 million by 2030. Another study also reported that 8.3% people suffer from DM to date with the number predicted to increase to more than 700 million globally by 2045.^2-4^ Apart from the use of insulin or oral hypoglycemic, other effective methods for treating diabetes have yet to be developed. Meanwhile, the initial symptom in the pathogenesis of diabetes is a decrease in the beta-cell function, which plays a role in causing type-2 diabetes mellitus (T2DM). Therefore, it is necessary to restore cell functions to prevent T2DM.^5^

Self-nanoemulsifying (SNE) is a method to improve the solubility and bioavailability of drugs, such as AND,^1^ lovastatin,^6^ nisoldipine,^7^ and glipizide.^8^ SNE has also been reported to be able to increase the anti-cancer activity of genistein^9^ and the antifungal activity of orally administered nystatin.^10^ Compared with the natural forms, solid SNE of polypeptide-k tested *in vivo* through oral administration to streptozotocin (STZ)-induced rats provided a better antidiabetic activity.^11^

AND is a bioactive compound found in the *Andrographis paniculata* Nees plant with antidiabetic^12^ and antioxidant activities.^13^ AND is poorly soluble in water, which is associated with low bioavailability in its oral administration.^1^ The administration of oral AND can lower plasma glucose levels in STZ-induced diabetic rats, but the activity of which depends on the dose administered^14^ and the treatment applied.^15^ A compound with low water solubility generally has limited absorption and bioavailability that is usually controlled by the dissolution rate of the drug in the gastrointestinal tract. Therefore, efforts need to be made to increase the solubility. In addition, low bioavailability influences the pharmacological effects of drug compounds.^16^ AND significantly impedes adipocyte differentiation induced by adipogenic agents and multiple daily doses of insulin (MDI) as well as inhibits adipogenesis-related transcription factor, peroxisome proliferator-activated receptor (PPAR), and the expression of PPARγ-targeted genes.^17^ AND has also been proven to raise glucose uptake depending on the time and dose in 3T3-L1 cells.^18^

The abovementioned data suggest that preparations in the form of SNE can improve the pharmacological effects of drug compounds. However, although ASNE preparation has been investigated to increase the bioavailability, studies of its antidiabetic activities have never been reported. This study therefore aimed to examine the SNE preparation of AND *in vivo* using rats with STZ-induced diabetes and *in vitro* using 3T3-L1 adipocyte cells.

## Materials and Methods

### Materials

AND was extracted and isolated from*Andrographis paniculata* collected from traditional markets in Yogyakarta (Indonesia) based on previous research.^15^ Capryol 90 was provided by Gattefose (Saint-Priest, France), Tween 20 and polyethylene glycol (PEG) 400 were obtained from Brataco (Yogyakarta, Indonesia), and STZ was purchased from Sigma-Aldrich (Missouri, USA). A colorimetric method (GOD-PAP) was used to measure glucose levels, and the antibodies used in the determination of the insulin expression were primary anti-insulin antibody from Santa Cruz Biotechnologies (California, USA) and secondary chicken anti-gout IgG antibody from Invitrogen (California, USA). Victoria Blue, hematoxylin, and eosin were purchased from Sigma Chemical Co. (California, USA), and 3T3-L1 preadipocytes were obtained from American Type Cell Culture Collection (Virginia, USA). Other materials included Dulbecco’s Modified Eagle’s Medium from Sigma (California, USA), fetal bovine serum and penicillin-streptomycin from Gibco (Maryland, USA), as well as 3-[4,5-dimethylthiazol-2-yl]-2,5-diphenyl-tetrazoliumbromide (MTT) from Calbiochem (Darmstadt, Germany).

### Preparation of AND-loaded SNE

SNE was prepared using Capryol 90 (oil phase), Tween 20 (surfactant), and PEG 400 (cosurfactant) at a ratio of 1:3:1. ASNE was obtained by mixing SNE with 15 mL of AND, based on a previously reported procedure.^1^

### Animal study

#### Animals

Twenty-four adult’s male Wistar rats with a body weight ranging from 200 g to 250 g at the beginning of the experiment were used in this current study. All of the experimental animals were placed in a standard-size cage at a temperature of 25°C under conditions of 12 h of light and 12 h of dark with the natural food and distilled water provided ad libitum.

#### Preparation of STZ-induced type 2 diabetic rats

Neonatal rats were administered with STZ dissolved in citrate buffer (pH 4.5) and injected intraperitoneally at a dose of 90 mg/kg BW, whereas neonatal control rats were given an intraperitoneal injection with the same amount of citrate buffer solution. Two and a half months after injection, STZ-induced rats were selected for the study by setting the levels of blood glucose before and after food administration. Rats with a glucose level of more than 180 mg/dL were considered to have diabetes.^13,19,20^

#### Experimental design

Pharmacological tests were performed on STZ-induced Wistar rats at birth, and after the age of 2.5 months, they were treated in line with the experimental design presented in Table 1. The medicine was administered for 8 days, and preprandial blood samples were taken on day 0, 2, 4, 6, and 8 after a 10 h fast. Two hours after feeding, blood samples were retaken to determine postprandial glucose levels.

**Table 1:** Experimental design of pharmacological test (N = 6)

**Groups**	**Animals**	**Treatment**
I	Control rats injected with 0.1 mL citrate buffer	Distilled water (vehicle) given twice a day orally
II	Normal rats	15 mg/mL AND suspension given twice a day orally
III	Neonatal rats induced by STZ	15 mg/mL ASNE given twice a day orally
IV	Neonatal rats induced by STZ	15 mg/mL AND suspension given twice a day orally

Abbreviations: STZ, streptozotocin; AND, andrographolide; ASNE, andrographolide in self-nanoemulsifying.

#### Determination of blood glucose levels

As previously explained, on day 0, 2, 4, and 8, preprandial blood samples were taken after a 10 h fast, and postprandial blood samples were taken 2 h after feeding. As much as 0.5 mL of blood was obtained through the eye vein (retro-orbital sampling). Serum was obtained after the centrifugation of blood samples at 3500 rpm for 15 min, and serum glucose was analyzed in spectrophotometry using the glucose oxidase-peroxidase method with a commercial biochemical diagnostic reagent kit (Glucose, GOD-PAP) (R1).^13,19,20^ The blank, standard, and samples were incubated at 37°C for 15 min, followed by the reading of solution absorbance using a UV/Vis spectrophotometer at a wavelength of 546 nm.

#### Histological observation of the islets of Langerhans and pancreatic beta cells

The rats were sacrificed at the end of the treatment. The pancreases of control rats and treated rats were taken and affixed in 10% formalin and phosphate buffer pH 7.5 for 24 h. The tissue was embedded in paraffin and sliced into sections of the desired thickness (4 μm), and the paraffin was then removed in xylene and dehydrated using a series of alcohol concentrations (70%, 80%, 90%, 95%, and 99%). The slices were then stained using hematoxylin and eosin (HE) or 1% Victoria Blue (pH 0.3) to evaluate the islets of Langerhans and pancreatic beta cells. The slides were cleaned with xylene, affixed using a mounting medium, and examined under a light microscope. Pancreatic beta cells were identified and detected in each slide to be analyzed.^13,19,20^ Preparation of the pancreas using HE staining was performed to observe the presence of pancreatic islets of Langerhans qualitatively. The pancreatic islets of Langerhans were quantitatively calculated to determine the average number of the islets of Langerhans. In addition, preparation of the pancreas with Victoria Blue staining was also performed to measure the average area of the islets of Langerhans and the percentage of pancreatic beta cells compared with the total number of cells in the islets of Langerhans.

#### Immunohistochemistry of pancreatic insulin

Another part of the pancreas was prepared with 4% formaldehyde in phosphate-buffered saline (PBS) for a minimum of 2 h. The tissue was dried in alcohol at a specific concentration and cleaned using a xylol cleaning agent. After being embedded in paraffin, 2–3 μm slices were obtained using a rotary microtome. The tissue was then attached to a slide, and the endogenous peroxidase activity in the tissue was inhibited using 3% H_2_O_2_ in methanol for 15 min, followed by washing with distilled water. One part of the tissue was then incubated with 20% horse serum for a minimum of 10 min to prevent non-specific binding. Another part of it was incubated at a room temperature with primary antibody to rat insulin by diluting it at 1:250 for 1 h and later with peroxidase-conjugated secondary antibody at 1:500 dilution for 1 h. The expression of pancreatic insulin could be visualized after incubation in a substrate for 15 min. The tissue was stained with hematoxylin for 30 min, dried with alcohol and xylol, and then affixed to a coverslip. The slides were then evaluated using a light microscope.^13,19,20^

#### In vitro study

#### Cell viability assay

Cell viability assay was performed using MTT^21^ with modification. The 3T3-L1 cells (Zen-Bio Inc., USA) were grown in Dulbecco’s modified Eagle’s medium (DMEM; Nacalai Tesque Inc., Japan) supplemented with 10% FBS and 1% antibiotics. Cells were seeded at a density of 2 × 10^5^ cells/mL and incubated at 37°C with 5% CO_2_. After reaching confluence, the cells were treated with samples and further incubated for 48 h. Once the treatment period was over, MTT solution (5 mg/mL) was added to each well, and the plate was incubated at 37°C with 5% CO_2_ for 4 h. Next, the supernatant was removed, and 100 µL of DMSO was added to each well after which spectrophotometric absorbance was measured at a wavelength of 570 nm with a reference wavelength of 630 nm. The 50% reduction in cell number relative to the control (IC_50_) was established by extrapolation from a graph of the experimental data.

#### Adipocyte differentiation (adipogenesis) assay

Cells were seeded at a density of 2 × 10^5^ cells/mL and incubated until they reached confluence. In two days of post-confluence (day 0), cells were induced to differentiate using an induction medium containing dexamethasone, 3-isobutyl-1-methylxanthine (IBMX), and insulin/samples (Table 2). Insulin treatment (1 µg/mL) was used as a control of the experiment. On day 2, the medium was changed into insulin and replaced every other day until day 8. After completing the differentiation course, cells were washed with PBS and fixed using 10% formalin for 1 h. Then, cells were rewashed with PBS and stained with Oil Red O for another hour. The cells were finally washed with distilled water and analyzed qualitatively using an inverted microscope (Leica, Germany). Meanwhile, quantitative analysis was performed by extracting the stain from the cells using isopropanol, and spectrophotometric absorbance was measured at 520 nm.^22^

**Table 2:** Adipocyte differentiation protocol

**Day**	**Treatment**	**Insulin**	**Untreated**
0	Complete DMEM Dexamethasone (0.25 µM) IBMX (0.5 mM) Samples of varying concentrations	Complete DMEM Dexamethasone (0.25 µM) IBMX (0.5 mM) Insulin (1 µg/mL)	Complete DMEM
2	Complete DMEM Insulin (1 µg/mL)	Complete DMEM Insulin (1 µg/mL)	Complete DMEM
4 – 8	Complete DMEM	Complete DMEM	Complete DMEM
10	Oil Red O Staining		

Abbreviations: DMEM, Dulbecco's modified Eagle's medium; IBMX, 3-isobutyl-1-methylxanthine.

### Statistical analysis

All statistical analyses were performed using Microsoft Office Excel 2010. The quantitative data calculations of HE and Victoria Blue staining, the percentage of beta cells with insulin expression, and adipocyte differentiation were expressed as mean ± SEM. One-way analysis of variance was used for the statistical analysis. A *P* value of less than 0.05 was considered as indicating a significant difference.

## Results and Discussion

### Animal study

#### Determination of blood glucose levels

**Figure 1:**
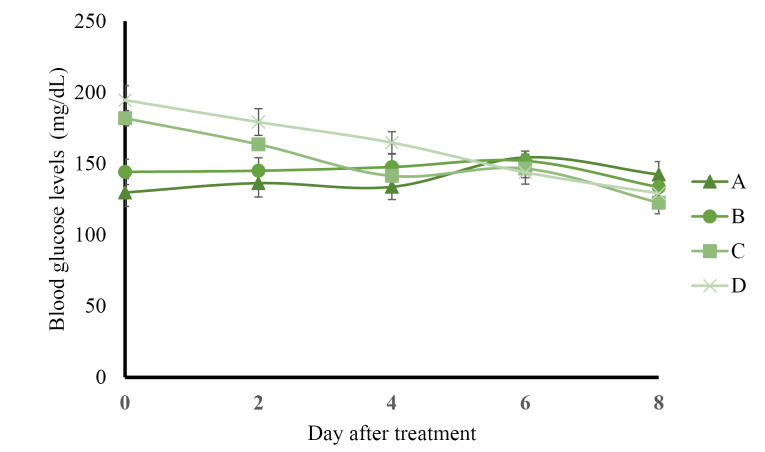
Preprandial blood glucose levels of the control rats (A), normal rats receiving andrographolide (AND) suspension (B), neonatal rats induced by streptozotocin (STZ) receiving andrographolide in self-nanoemulsifying, ASNE (C), and neonatal rats induced by STZ receiving AND suspensions (D). The variations of the average are shown as SD, with three times repetition.

**Figure 2:**
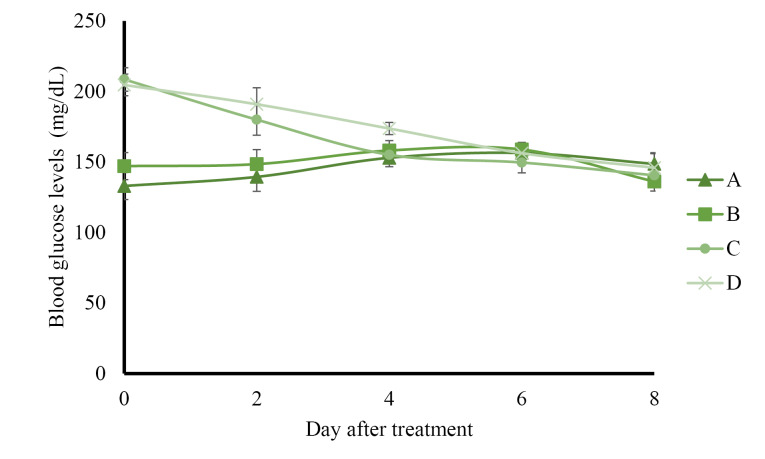
Postprandial blood glucose levels of the control rats (A), normal rats receiving andrographolide (AND) suspension (B), neonatal rats induced by streptozotocin (STZ) receiving andrographolide in self-nanoemulsifying, ASNE (C), and neonatal rats induced by STZ receiving AND suspension (D). The variations of the average are shown as SD, with three times repetition.

The results of preprandial blood glucose level measurement presented in Figure 1 show that control rats administered with solvents and normal rats administered with AND suspension had normal blood glucose levels (less than 160 mg/dL) during 8 days of treatment. The administration of ASNE reduced the blood glucose levels to normal on day 4, whereas AND suspension decreased the blood glucose levels to normal on day 6. The postprandial blood glucose levels described in Figure 2 indicate the same results as those in the preprandial in terms of the normalization of blood glucose levels, which occurred on day 4 in ASNE and day 6 in AND suspension.

Hyperglycemia induced by STZ has been widely used as an experimental model to study hypoglycemic activities. The mechanism of diabetes caused by STZ involves the selective destruction of pancreatic beta cells that causes hypoinsulinemia, thus resulting in hyperglycemia and reduced glucose uptake as the main characteristics of the disease.^23,24^ ASNE could reduce the postprandial blood glucose levels on days 2, 4, 6, and 8 compared with AND suspension (*P*  < 0.05) as shown in Figure 2. In general, ASNE could provide a more immediate effect to normalize blood glucose levels compared with AND suspension. The pharmacokinetic profile supports the results of blood glucose level measurement as previously reported^1^ in which the time taken by ASNE to reach the maximum drug concentration in blood (T_max_) was 1.72-fold faster than that by AND suspension. Therefore, the pharmacological effects of ASNE are more immediate than those of AND suspension. A study of the correlation between the pharmacokinetic and pharmacological effects on diabetes has also proven that insulin self-nano emulsifying drug delivery system (SNEDDS) produced 2.7- and 3.4-fold enhancement in the relative bioavailability and glucose reduction.^25^ Glipizide solid SNEDDS formulation showed increases in C_max_ (3.4-fold) and AUC_0-12h_ (2.7-fold) and a decrease in the plasma glucose level by 1.3-, 1.3-, and 2.9-fold compared with the pure drug.^26^

#### Histological observation of the islets of Langerhans and pancreatic beta cells

**Figure 3:**
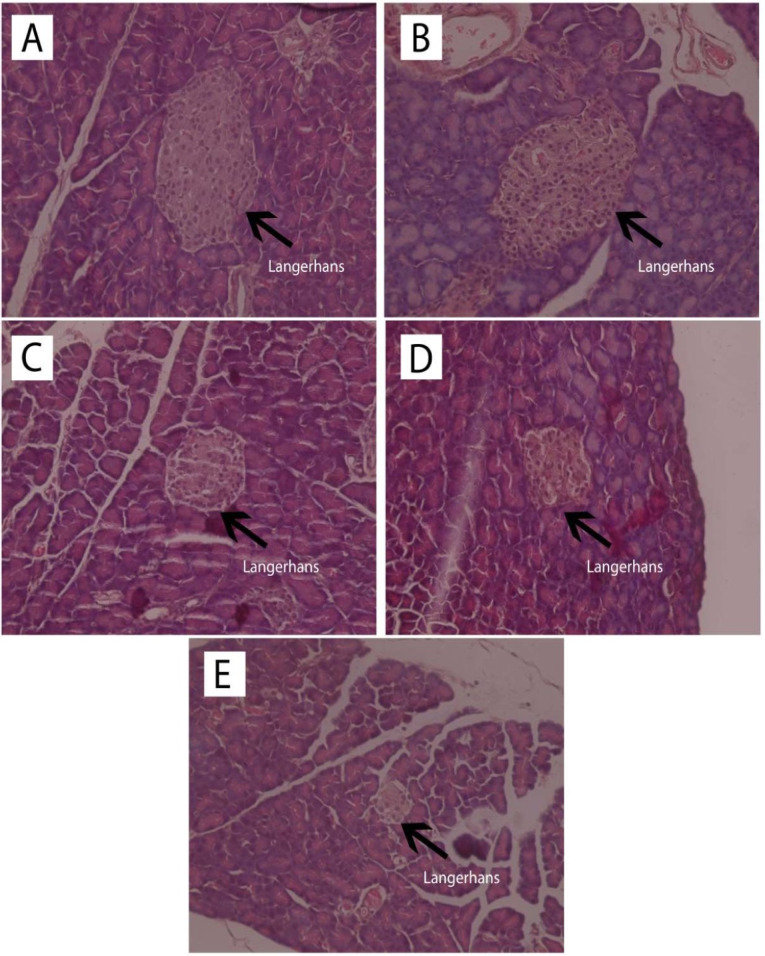
Observation of the islets of Langerhans (L) with HE staining (400x magnification) of the normal rats (A), normal rats receiving andrographolide (AND) suspension (B), diabetic rats receiving andrographolide in self-nanoemulsifying, ASNE (C), diabetic rats receiving AND suspension (D), and diabetic rats as the negative control (E).

**Figure 4:**
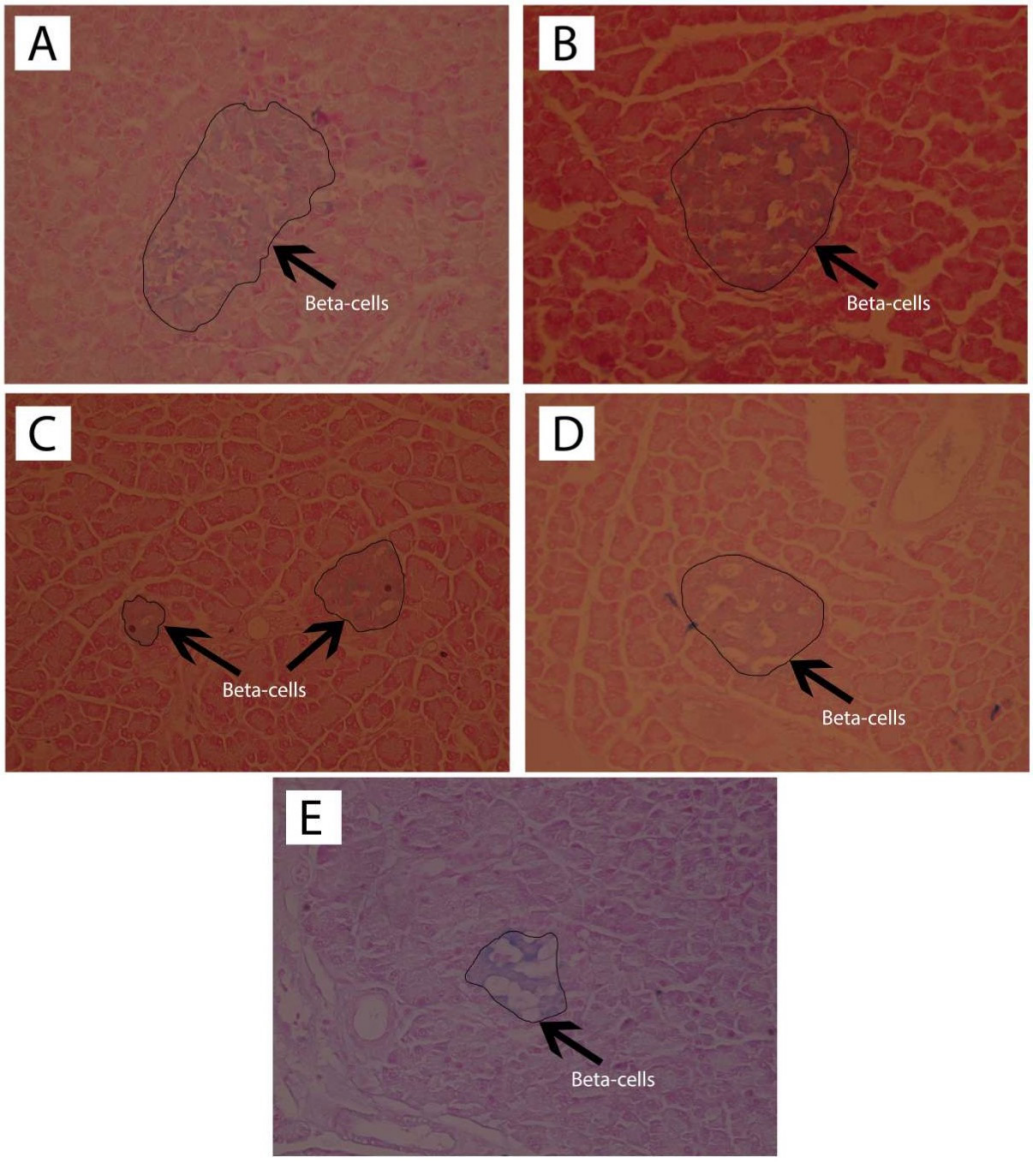
Observation of the pancreatic beta cells with Victoria Blue staining (400x magnification) of the normal rats (A), normal rats receiving andrographolide (AND) suspension (B), diabetic rats receiving andrographolide in self-nanoemulsifying, ASNE (C), diabetic rats receiving AND suspension (D), and diabetic rats as the negative control (E).

Observation of the islets of Langerhans was carried out with HE staining, and pancreatic beta cell observation used the Victoria Blue staining. The results of histological observation of the islets of Langerhans with HE staining are shown in Figure 3 while the histopathological findings of pancreatic beta cells with Victoria Blue staining are presented in Figure 4.

Quantitative calculations from the HE and Victoria Blue staining for the number of the islets of Langerhans, the area of the islets of Langerhans, and the percentage of pancreatic beta cells compared with the total number of cells in the islets of Langerhans are presented in Table 3.

**Table 3:** Quantitative calculations in Hematoxylin-Eosin (HE) and Victoria Blue staining to count the number of the islets of Langerhans, the area of the islets of Langerhans, and the percentage of pancreatic beta cells (n = 3)

**Code**	**Treatment**	**The number of islets of Langerhans**	**The area of islets of Langerhans (µm** ^ 2 ^ **)**	**The percentage of pancreatic beta cells (%)**
A	Normal rats	9.00 ± 1.41^*^	868,761 ± 135.20^*^	69.83 ± 1.79^*^
B	Normal rats receiving AND suspension	7.50 ± 1.29^*^	623,046 ± 13.99^*^	66.12 ± 8.6^*^
C	Diabetic rats receiving ASNE	5.75 ± 2.36^*^	387,161 ± 64.00^*^	61.70 ± 1.12^*^
D	Diabetic rats receiving AND suspension	5.25 ± 1.50^*^	681,929 ± 157.41^*^	65.49 ± 0.48^*^
E	Diabetic rats as the negative control	2.75 ± 1.26	274,307 ± 29.7	40.52 ± 4.56^*^

^*^ Significantly different from the negative control (*P*  < 0.05) Abbreviations: AND, andrographolide; ASNE, andrographolide in self-nanoemulsifying.

Table 3 showed that ASNE (C) and AND suspension (D) were associated with different numbers of the islets of Langerhans, 5.75 ± 2.36 and 5.25 ± 1.50, respectively (*P*  < 0.05), whereas in the initial condition (negative control, E), the number was 2.75 ± 1.26. The area of the islets of Langerhans also increased (*P*  < 0.05) from 274.307 ± 29.7 µm in the negative control (E) to 387.161 ± 64.00 and 681.929 157.41 µm in ASNE (C) and AND suspension (D), respectively. These findings prove that ASNE and AND suspension could increase the number and area of the islets of Langerhans. They were also able to increase the percentage of beta cells (*P*  < 0.05) from 40.52 ± 4.56% in the negative control (E) to 61.70 ± 1.12% and 65.49 ± 0.48%, respectively.

The area of the islets of Langerhans and the percentage of pancreatic beta cells in AND suspension (D) are higher than in ASNE (C) as presented in Table 3. AND suspension is prepared by dissolving AND in a suspending agent while ASNE is produced by dissolving AND in an oil, surfactant and cosurfactant as the vehicle, but ASNE will spontaneously disperse when dissolved in water. ASNE plays a role in accelerating the repair effect on the islets of Langerhans and pancreatic beta cells. This finding showed that AND suspension could improve the area of the islets of Langerhans and pancreatic beta cells better than ASNE. However, the effects remain the same as both can produce pancreatic beta cells, normally, with the percentage exceeds 60%.

The number of beta cells determines the area of the Islets of Langerhans in that location. Rupture of the Islets of Langerhans and decreased function of pancreatic beta-cells cause diabetes. In the abovementioned model, STZ reaches beta cells through a mechanism of glucose transport. STZ has been reported to cause DNA alkylation by releasing high concentrations of nitric oxide and nitrosourea, thus causing aconitase inhibition. Insulin resistance or insulin deficiency (T2DM) in animal models induced by STZ depends on several factors, such as STZ dose, animal age, and animal strain.^19,27^ The use of STZ-induced type-2 diabetic experimental animals, such as neonatal rats aged 2 days, with damaged pancreatic beta cells through regeneration remains possible. Rats experience hyperglycemia due to insulin deficiency.

The presence of DM in experimental animals will be observable during their selection for treatment based on the blood glucose levels that exceed the average. The maximum glucose level of normal rats is 160 mg/dL, whereas the selected rats with DM had a blood glucose level of more than 180 mg/dL. Similarly, in the experiments with indolizine derivatives and capsaicinoids, these compounds lowered blood glucose levels in STZ-induced diabetic rats while also selectively damaging pancreatic beta cells via reactive oxygen species. The administration of STZ to rats will damage the pancreatic necrosis of the beta cells in the islets of Langerhans and also impair the function of the pancreas. This condition ultimately results in diabetes, which causes an increase in plasma glucose levels (because it decreases plasma insulin) and weight loss.^28,29^ It was also reported that the mechanism of the antidiabetic activity of a natural compound named corilagin occurs via increased utilization of peripheral glucose by skeletal muscles apart from a beta-cell stimulation.^30^

The improvement of insulin sensitivity is a critical approach in the management of T2DM; it was reported that AND derivatives ameliorate insulin sensitivity in rats.^31^ Observation of the islets of Langerhans using HE staining also showed that in normal rats (A) and normal rats given AND suspension (B) without induced STZ, the islets of Langerhans appeared normal, without inflammation, degeneration, or necrosis. Diabetic rats given ASNE (C), and AND suspension (D) showed normal islets of Langerhans and no inflammation, congestion, degeneration, or necrosis (Figure 3). In untreated diabetic rats (E), the islets of Langerhans were rare, atrophic, and relatively small in size, whereas some cells in them were normal while some exhibited degeneration and necrosis (Figure 3). The data presented above demonstrate that AND provided a repairing effect for the islets of Langerhans in the pancreas of rats.

It has been proven that ASNE and AND suspension could reduce blood glucose levels through improvements in pancreatic insulin, but ASNE had a more immediate effect than AND suspension. STZ-induced diabetic rats had significantly decreased blood glucose levels with the administration of ASNE and AND suspension. Compared with AND suspension, ASNE showed a faster process in decreasing blood glucose levels. These findings are likely related to the higher bioavailability of ASNE compared with that of AND suspension in terms of the shorter time to achieve maximum drug concentration in blood (T_max_). The blood-glucose-lowering effect of ASNE and AND suspension is likely caused by the stimulation of beta cells in the islets of Langerhans, which results in more insulin release. Increased plasma insulin levels reinforce this finding in diabetic rats treated with ASNE and AND suspension, and there is also an increase in the number of immunoreactive cells that secrete insulin in many parts of the pancreatic islets as indicated by the immunohistochemical analysis.^23^ Another possible mechanism of blood glucose level reduction is through potentiating the effects of plasma insulin by increasing pancreatic insulin secretion from the cells of the islets of Langerhans or their release from the bonds.^32^

#### Immunohistochemistry of pancreatic beta cells

**Figure 5:**
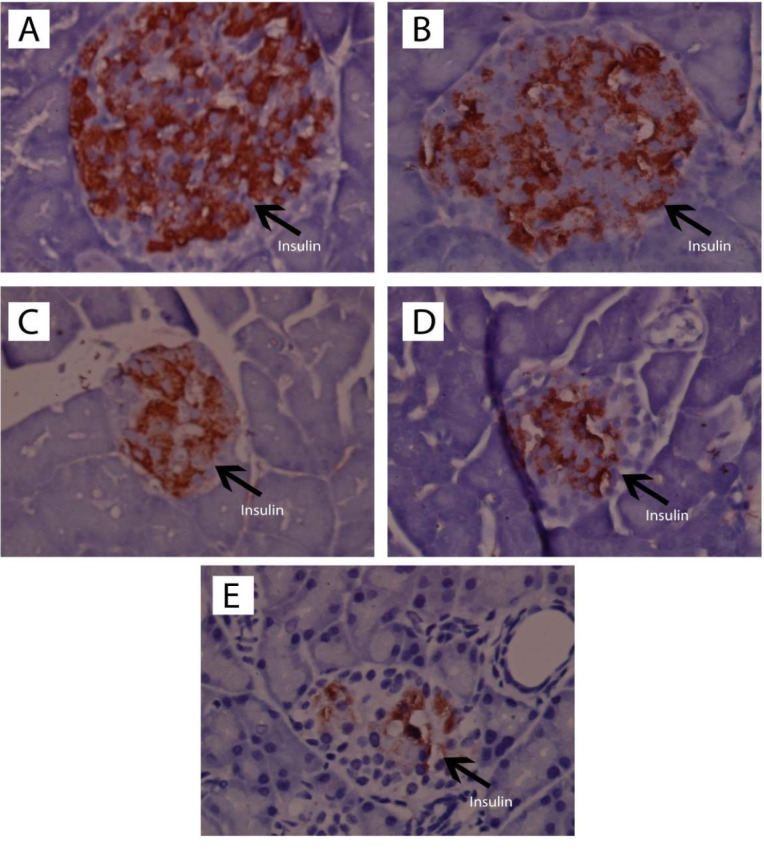
Observation of immunohistochemical-stained section insulin pancreas (I) (1000x magnification) of the normal rats (A), normal rats receiving andrographolide (AND) suspension (B), diabetic rats receiving andrographolide in self-nanoemulsifying, ASNE (C), diabetic rats receiving AND suspension (D), and diabetic rats as the negative control (E).

The staining process for immunohistochemistry testing followed the preparation method for HE staining blocks, and observation of insulin expression on the islets of Langerhans was performed using insulin antibodies. The results of pancreatic insulin immunohistochemical analysis are presented in Figure 5.

Preparation of the pancreas with immunohistochemistry of insulin antibodies was performed with the aim of counting the beta-cells with brown cytoplasm, which showed insulin expression, in comparison with all of the non-brown cells in the islets of Langerhans. The results of the quantitative immunohistochemical analysis are presented in Table 4.

**Table 4:** The number and percentage of beta-cells with insulin expression compared with other cells in the islets of Langerhans (n = 3)

**Code**	**Treatment**	**The number insulin of expression**	**The percentage of insulin expression (%)**
A	Normal rats	79.40 ± 2.55^*^	78.06 ± 3.09^*^
B	Normal rats receiving AND suspension	74.98 ± 8.41^*^	74.98 ± 1.73^*^
C	Diabetic rats receiving ASNE	47.70 ± 5.52^*^	60.43 ± 2.56^*^
D	Diabetic rats receiving AND suspension	41.50 ± 6.36^*^	39.05 ± 1.02^*^
E	Diabetic rats as the negative control	14.70 ± 4.95	15.52 ± 5.77

^*^Significantly different from the negative control (*P*  < 0.05). Abbreviations: AND, andrographolide; ASNE, andrographolide in self-nanoemulsifying.

Table 4 showed that ASNE (C) and AND suspension (D) could increase (*P*  < 0.05) the number of cells with insulin expression compared with the negative control (E), initially from 14.70 ± 4.95 to 47.70 ± 5.52 and 41.50 6.36. Other cells also showed that ASNE (C) and AND suspension (D) could also increase their percentages (*P*  < 0.05) compared with the negative control (E), from 15.52 ± 5.77% to 60.43 ± 2.56% and 39.05 1.02%.

Pancreatic histopathological investigation in diabetic rats induced by STZ showed histological changes in the pancreas. This damage occurs because STZ attacks the pancreatic beta cells through glucose transporter 2, which results from the damage of beta cells by DNA alkylation. The production of superoxide radicals also causes this damage with the help of xanthine oxidase and free radicals of nitric oxide. Free radicals make a significant contribution to the development of DM as such, which causes damage to beta cells.^33,34^ Treatment with AND may protect the cells from free radicals caused by STZ. Zhang reported that AND increases insulin and prevents the loss of beta cells and their dysfunction in soleus muscles.^35^

#### In vitro study

#### Effect of SNEEDS on adipocyte differentiation

**Figure 6:**
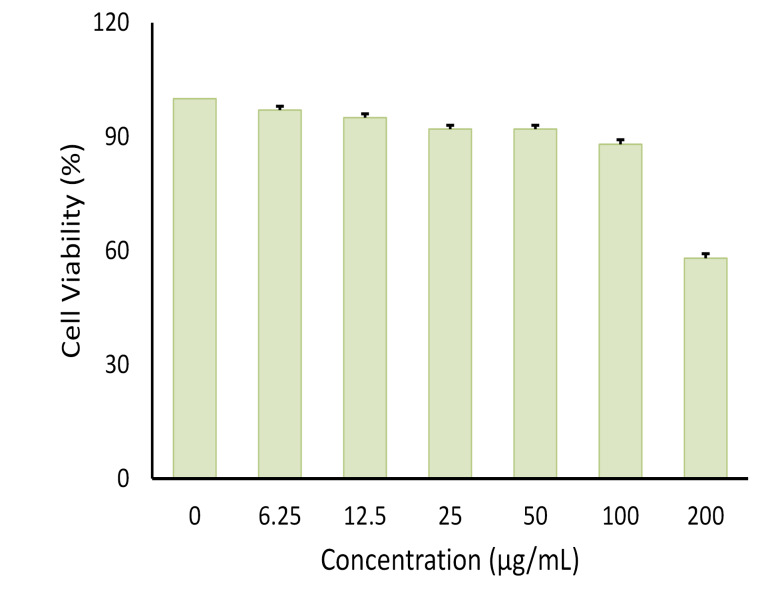
Effect of different concentrations of andrographolide in self-nanoemulsifying (ASNE) on cell viability of 3T3-L1 preadipocytes. (Data represented as means ± SE of three independent experiments).

**Figure 7:**
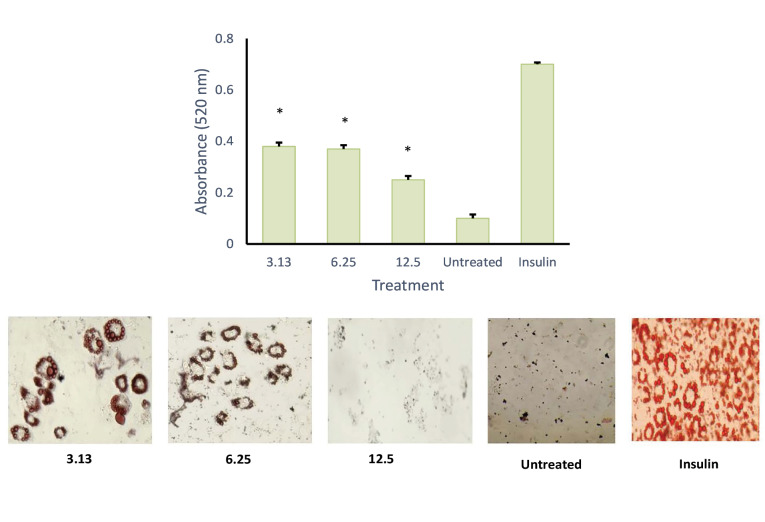
Effect of andrographolide, AND (3.13, 6.25 and 12.50 µg/mL) on adipocyte differentiation of 3T3-L1 cells, quantification of lipid (top) and intracellular lipid droplet and total lipid content with 400x magnification (bottom). *P  < 0.05 when compared with the positive control

**Figure 8:**
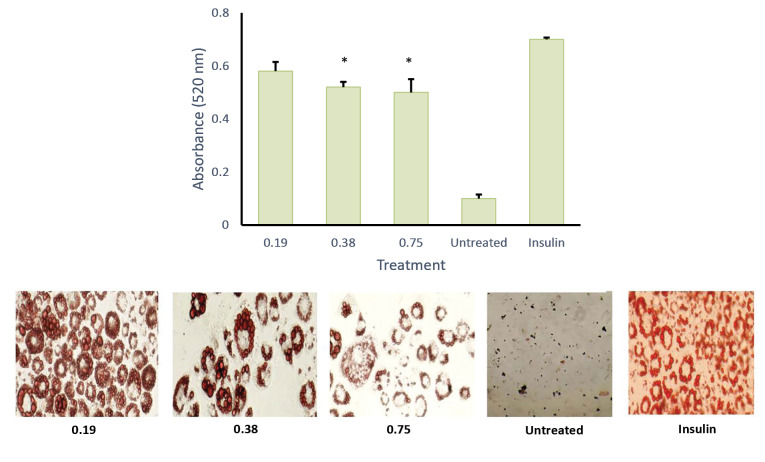
Effect of andrographolide in self-nanoemulsifying, ASNE (0.19, 0.38 and 0.75 µg/mL) on adipocyte differentiation of 3T3-L1 cells, quantification of lipid (top) and intracellular lipid droplet and total lipid content with 400x magnification (bottom). * P  < 0.05 when compared with the positive control

MTT assay was conducted to evaluate the cytotoxic activity of the emulsion on cultured cells (Figure 6). It was found that, at 100 µg/mL, the emulsion had no cytotoxic effect on the cells. This data was used as the baseline concentration for the adipocyte differentiation assay. As shown in Figures 7 and 8, both AND suspension and ASNE inhibited the formation of mature adipocytes in a dose-dependent manner.

To understand the critical stage of adipogenesis inhibition by AND suspension (Figure 7), the preparation was added to the culture medium in 2 days of post-confluence (day 0), and cells were induced to differentiate using an induction medium containing dexamethasone, IBMX, and insulin/or AND (3.13, 6.25 and 12.5 μg/mL). On day 2, the medium was changed into an insulin medium and replaced every 2 days until day 8. After completing the differentiation course, cells were washed with PBS and fixed using 10% formalin for 1 h. Then, cells were rewashed with PBS and stained with Oil Red O for another hour. Using the same method (Figure 8), ASNE preparation was added in 2 days of post-confluence (day 0), and cells were induced to differentiate using an induction medium containing dexamethasone, IBMX, and insulin/or ASNE (0.19, 0.38 and 0.75 μg/mL). This finding showed that the anti-adipogenic effect of ASNE at a low dose (0.75 μg/mL) is similar to that of AND suspension at a dose of 3.13 μg/mL, thus indicating that ASNE is approximately 4 times more effective than AND suspension.

In the *in vitro* study, AND tended to have an anti-obesity effect on 3T3-L1 adipocytes. Another study previously showed thatAND significantly inhibited the differentiation of preadipocytes, which was induced by adipogenic agents and MDI.^36^ Adipose tissue dysfunction causes metabolic syndrome involving insulin resistance and type-2 diabetes. Adipose is not only known for its capacity to store excess food energy in the form of triglycerides but has also been recognized as playing an essential role in the control of energy metabolism. Meanwhile, adipocytes are cells that can save excess energy in the form of triglycerides, and their level of differentiation is closely related to obesity.^22,37,38^ Adipocytes become the main drug target for diabetes and metabolic syndrome caused by obesity. Obesity itself is caused by the process of fat accumulation which can be divided into two main stages: the process of differentiation of adipocytes (adipogenesis) and the process of fat synthesis (lipogenesis).

Inhibition is related to a transcription factor, PPARγ, as well as the expression of PPARγ-targeted genes. When comparing the effectiveness of ASNE and AND in the *in vitro* study, it was found that ASNE delivery was twice more effective than AND. However, AND also increased the glucose uptake in a time- and dose-dependent manner in 3T3-L1 cells.^17^

## Conclusion

ASNE formulation could reduce blood glucose levels to normal in STZ-induced rats in a faster and more effective manner than AND suspension. The decreased blood glucose levels to normal occurred on day 4 in ASNE and day 6 in AND suspension. Histological tests showed that ASNE could regenerate pancreatic beta cells and increase insulin expression. In the adipocyte differentiation assay, it was demonstrated that the ASNE formulation achieved better delivery of AND.

## Ethical Issues

This study received an approval from the Animal Care Committee of Gadjah Mada University, Yogyakarta, Indonesia (No. 412/KEC-LPPT/XII/2015), and was performed by referring to the European Community guidelines for studies on experimental animals.

## Conflict of Interest

The authors declare no conflict of interest.

## Funding

The project was funded by Islamic University of Indonesia under the Fellowship Program No. 1982.C/Rek/40/WR.I/VII/2019 .
